# Draft Genome Sequence of Mn(II)-Oxidizing Pseudomonas resinovorans Strain MO-1

**DOI:** 10.1128/genomeA.00088-18

**Published:** 2018-03-15

**Authors:** Shuji Matsushita, Michiharu Nakano, Yoshiteru Aoi, Tomonori Kindaichi, Noriatsu Ozaki, Akiyoshi Ohashi

**Affiliations:** a Department of Civil and Environmental Engineering, Graduate School of Engineering, Hiroshima University, Higashi-Hiroshima, Hiroshima, Japan; b Western Region Industrial Research Center, Hiroshima Prefectural Technology Research Institute, Kure, Hiroshima, Japan; c Laboratory of Plant Chromosome and Gene Stock, Graduate School of Science, Hiroshima University, Higashi-Hiroshima, Hiroshima, Japan; d Department of Molecular Biotechnology, Graduate School of Advanced Science of Matter, Hiroshima University, Higashi-Hiroshima, Hiroshima, Japan

## Abstract

Pseudomonas resinovorans strain MO-1, which possesses a high ability to oxidize Mn(II), has been isolated from oligotrophic pond sediment. The draft genome sequence consists of 6,252,942 bp and has a G+C content of 63.4%. Strain MO-1 has 5,694 coding sequences, including 13 putative Mn(II) oxidation genes.

## GENOME ANNOUNCEMENT

Mn(II)-oxidizing bacteria (MnOB) produce biogenic Mn(III, IV) oxides (Bio-MnO_x_) in a wide variety of environments (1). The produced Bio-MnO_x_ is abundant in natural environments and adsorbs metal ions and/or oxidizes metals (2), thereby contributing to the oxidation of organic matter (3, 4). Phylogenetically diverse MnOB species exist, many with differing Mn(II) oxidation abilities (5). However, the oxidation mechanisms are still not fully elucidated. Additionally, why MnOB oxidize Mn(II) at all remains unclear (6). The isolation and characterization of diverse MnOB are essential for solving these questions. We isolated and screened more than 50 strains of MnOB from various environments and identified the strain with the highest Mn(II) oxidation ability from a manganese- and iron-rich sediment in an oligotrophic pond (34°39.891′N, 132°71.226′E). We named this bacterium MO-1; it is a Pseudomonas resinovorans strain, yet surprisingly, it has not been confirmed as a MnOB. Here, we present the draft genome sequence and putative Mn(II) oxidation genes of strain MO-1.

Strain MO-1 genomic DNA was extracted using a commercial kit (NucleoSpin tissue kit; Macherey-Nagel), according to the manufacturer’s protocol. The extracted DNA was sequenced using a 101-bp paired-end sequencing method with an Illumina HiSeq 2500 platform at Hokkaido System Science Co., Ltd. (Sapporo, Japan), obtaining 22,074,708 reads, with approximately 350-fold genome coverage. After the adaptors were trimmed using the Trimmomatic program version 0.36 (7), the cleaned sequence reads were assembled using the Platanus program version 1.2.4 (8). We obtained 122 contigs by removing short contigs of less than 300 bp, resulting in a draft genome of 6,252,942 bp, with a G+C content of 63.4%. The longest contig has 314,004 bp, and the calculated *N*_50_ length is 110,654 bp. The draft genome was annotated using the Microbial Genome Annotation Pipeline (MiGAP) version 1.060 (http://www.migap.org/) and was estimated to have 5,694 coding sequences (CDSs), including 2 rRNA genes and 61 tRNA genes.

Two gene families encoding multicopper oxidase (MCO) and heme peroxidase oxidase domains play an important role in Mn(II) oxidation in several MnOB. Of our 5,694 CDSs, 1, 8, and 4 CDSs were identified as being related to Mn(II) oxidation; these were homologous to *mnxG* (locus tag PputGB1_2447 under GenBank accession no. CP000926), *mcoA* (locus tag PputGB1_2665 under GenBank accession no. CP000926) and *mopA* (locus tag PputGB1_3353 under GenBank accession no. CP000926) of Pseudomonas putida GB-1 (9, 10), respectively, using BLASTP analysis (E value ≤ e^−50^) (11). The highest similarities between our sequences and those P. putida sequences calculated by the Needleman-Wunsch global alignment algorithm (12) are 82.2, 67.3, and 56.7% for *mnxG*, *mcoA*, and *mopA*, respectively. Strain MO-1 possesses 13 putative Mn(II) oxidation genes, much more than the 3 annotated Mn(II) oxidation genes in P. putida GB-1.

### Accession number(s).

The P. resinovorans strain MO-1 genome sequence has been deposited in DDBJ/EMBL/GenBank under the accession no. BDMA00000000. The version described in this paper is the first version, BDMA01000000.
